# An Evaluation of Digital Health Tools for Diabetes Self-Management in Hispanic Adults: Exploratory Study

**DOI:** 10.2196/12936

**Published:** 2019-07-16

**Authors:** Leah Yingling, Nancy A Allen, Michelle L Litchman, Vanessa Colicchio, Bryan S Gibson

**Affiliations:** 1 Department of Biomedical Informatics University of Utah School of Medicine Salt Lake City, UT United States; 2 College of Nursing University of Utah School of Medicine Salt Lake City, UT United States

**Keywords:** type 2 diabetes, Hispanic, blood glucose self-monitoring, culturally appropriate technology, mobile app

## Abstract

**Background:**

Although multiple self-monitoring technologies for type 2 diabetes mellitus (T2DM) show promise for improving T2DM self-care behaviors and clinical outcomes, they have been understudied in Hispanic adult populations who suffer disproportionately from T2DM.

**Objective:**

The objective of this study was to evaluate the acceptability, feasibility, and potential integration of wearable sensors for diabetes self-monitoring among Hispanic adults with self-reported T2DM.

**Methods:**

We conducted a pilot study of T2DM self-monitoring technologies among Hispanic adults with self-reported T2DM. Participants (n=21) received a real-time continuous glucose monitor (RT-CGM), a wrist-worn physical activity (PA) tracker, and a tablet-based digital food diary to self-monitor blood glucose, PA, and food intake, respectively, for 1 week. The RT-CGM captured viewable blood glucose concentration (mg/dL) and PA trackers collected accelerometer-based data, viewable on the device or an associated tablet app. After 1 week of use, we conducted a semistructured interview with each participant to understand experiences and thoughts on integration of the data from the devices into a technology-facilitated T2DM self-management intervention. We also conducted a brief written questionnaire to understand participants’ self-reported T2DM history and past experience using digital health tools for T2DM self-management. Feasibility was measured by device utilization and objective RT-CGM, PA tracker, and diet logging data. Acceptability and potential integration were evaluated through thematic analysis of verbatim interview transcripts.

**Results:**

Participants (n=21, 76% female, 50.4 [SD 11] years) had a mean self-reported hemoglobin A_1c_ of 7.4 [SD 1.8] mg/dL and had been diagnosed with T2DM for 7.4 [SD 5.2] years (range: 1-16 years). Most (89%) were treated with oral medications, whereas the others self-managed through diet and exercise. Nearly all participants (n=20) used both the RT-CGM and PA tracker, and 52% (11/21) logged at least one meal, with 33% (7/21) logging meals for 4 or more days. Of the 8 possible days, PA data were recorded for 7.1 [SD 1.8] days (range: 2-8), and participants averaged 7822 [SD 3984] steps per day. Interview transcripts revealed that participants felt most positive about the RT-CGM as it unveiled previously unknown relationships between lifestyle and health and contributed to changes in T2DM-related thoughts and behaviors. Participants felt generally positive about incorporating the wearable sensors and mobile apps into a future intervention if support were provided by a health coach or health care provider, device training were provided, apps were tailored to their language and culture, and content were both actionable and delivered on a single platform.

**Conclusions:**

Sensor-based tools for facilitating T2DM self-monitoring appear to be a feasible and acceptable technology among low-income Hispanic adults. We identified barriers to acceptability and highlighted preferences for wearable sensor integration in a community-based intervention. These findings have implications for the design of T2DM interventions targeting Hispanic adults.

## Introduction

### Type 2 Diabetes Among Hispanic Adults in the United States

Type 2 diabetes mellitus (T2DM) disproportionately affects racial and ethnic minorities and poses a significant risk of morbidity and early mortality [1]. Hispanic adults with T2DM, for instance, have higher hemoglobin A_1c_ (HbA_1c_) levels and rates of T2DM-related complications compared with non-Hispanic white adults [2,3]. Such disparities may be attributed to the unique barriers Hispanic adults face in achieving optimal T2DM management, including lower rates of health insurance coverage and language and literacy challenges [4].

### Diabetes Self-Management in Hispanic Adults

Effective long-term management of T2DM can be achieved with dedicated patient self-management, where individuals are actively engaged in their health-related behaviors and decisions. Components of T2DM self-management include physical activity (PA), tracking food intake and blood glucose levels, and taking medications, among others [5]. Hispanic adults with T2DM have difficulties engaging in T2DM self-management activities compared with non-Hispanic whites, and many struggle to meet the American Diabetes Association’s self-management recommendations [6].

### Technology-Facilitated Type 2 Diabetes Mellitus Self-Management

Wearable sensors and mobile apps have shown promise for capturing both self-reported and objective measures of T2DM self-management (ie, continuous blood glucose levels, food intake, and PA) in the context of a patient’s daily life. Although these devices are often explored separately [7], integrating data from multiple devices and data sources provides the potential to yield new knowledge and illuminate relationships between multiple health behaviors (eg, the effect of PA on blood glucose levels). Understanding the facilitators and barriers to using multiple self-monitoring tools for self-management may reveal opportunities to improve T2DM self-care behaviors and clinical outcomes, particularly for patient populations who suffer disproportionately from T2DM.

In this community-based participatory research (CBPR) pilot study, we assessed the minimally guided use of multiple digital health tools for T2DM self-monitoring by a Hispanic community-based population. We (1) evaluated the use of wearable sensors and mobile apps for capturing T2DM self-management behaviors; (2) investigated the facilitators and barriers of implementing technology-facilitated T2DM self-management interventions in a low socioeconomic status Hispanic community; and (3) identified opportunities for integrating these tools and their data into a future self-management intervention tailored to low-income Hispanic communities.

The purpose of this study was to evaluate the acceptability, feasibility and potential integration of wearable sensors and mobile apps for T2DM self-management among a Hispanic community-based population with self-reported T2DM.

## Methods

### Study Background

This study was part of a large 3-tiered Patient Centered Outcomes Research Institute–funded project. In tier I, a community advisory board (CAB) of Hispanic adults with T2DM was developed to identify potential technology solutions that would be useful and acceptable to a Hispanic population. In tier II, a larger sample of Hispanic adults with T2DM was surveyed about technology-related solutions that could support T2DM. This CBPR study was conducted following the survey to pilot test 3 different self-monitoring technologies and understand facilitators and barriers to use among Hispanic adults with self-reported T2DM. The University of Utah Institutional Review Board approved this study. All participants provided written informed consent.

### Study Population

Participants were recruited from August 2016 to September 2017 through outreach performed by members of our CAB and through community partnerships. Recruitment fliers were also distributed at local health clinics and with our nonprofit partners. Participants were eligible for this study if they were aged 19 to 85 years, had been diagnosed with T2DM, were willing to avoid acetaminophen during the study period because of its interaction with the RT-CGM sensor, provided informed consent, and possessed sufficient English language proficiency to carry out study tasks. Our study protocol did not require that participants have previous experience with technology. With this inclusion criteria, we aimed to recruit a diverse range of Hispanic adults with T2DM to reflect various types of technology users.

### Study Design

At study initiation, participants received 3 devices for T2DM self-monitoring: a Dexcom G4 real-time Continuous Glucose Monitor (RT-CGM; Dexcom Inc), Fitbit Surge (Fitbit), and an iPad (Apple) preloaded with health-related mobile apps, including Headspace, Fitbit, HealthWatch 360, EsTuDiabetes, Diabetes Detective, and Fooducate (see Multimedia Appendix 1 for brief descriptions). Participants also received a blood glucose meter to collect finger-stick blood glucose measurements twice daily, required for RT-CGM calibration.

The wrist-worn PA monitor, a Fitbit Surge, collected accelerometer-based data on the amount and intensity of PA (eg, steps taken, calories burned, distance travelled, and floors climbed). The RT-CGM, a Dexcom G4 Platinum Professional Glucose Monitoring System, collected real-time continuous sensor glucose readings every 5 min during a sensor session (7 days) and communicated the reading and trends to the patient. The system consists of 3 parts: a sensor, receiver, and transmitter. The sensor is inserted in the patient’s subcutaneous tissue; this was performed by the study nurse. The transmitter, a gray chip connected to the sensor pod, communicates glucose readings between the sensor and the receiver; the receiver, a small handheld device, displays sensor glucose readings, trends, and direction and rate of glucose change. The tablet, an iPad, was preloaded with various T2DM self-monitoring and educational apps by the research team. The research team prepurchased a data plan for the iPad at study onset to allow for the use of the iPad apps when a wireless connection was unavailable to participants.

Participants were instructed to use the RT-CGM and activity tracker continuously (but unguided) for 7 days. They were also instructed to log their food intake using the Fitbit or HealthWatch 360 apps and were invited to explore other apps included on the iPad as needed or desired. A 30-min one-on-one training session explaining this protocol and specific instructions on device use (ie, calibrating the RT-CGM, self-monitoring blood glucose, logging meals on the iPad, and charging the devices) was conducted on the day of device distribution by a member of the research team. The industry instruction manual for each device (eg, Fitbit and Dexcom G4) and a device list were also provided to all participants at this time, both of which were written in English only (see Multimedia Appendix 2). Participants were also provided with the research team’s contact information in case any questions arise.

After 1 week of T2DM self-monitoring (a testing period determined by the 7-day lifespan of the RT-CGM sensor) with the 3 devices, a semistructured interview (Multimedia Appendix 3) was conducted by a bilingual study nurse (to ensure questions were understood and accurately interpreted by participants) and a clinical informaticist. Participants also completed a questionnaire that captured sociodemographic information, T2DM-related information, and technology usage and preferences.

The outcomes of interest in our study were (1) feasibility of the wearable activity tracker and RT-CGM, as measured by days with self-directed device use and (2) acceptability of the system as measured by the results of semistructured interviews designed to elicit participants’ feedback on the devices as standalone tools and prompt their suggestions for potential design improvements and opportunities for incorporating these tools in an integrated T2DM self-management intervention.

### Data Collection

During the 7-day monitoring period, participants synchronized their Fitbit device with the Fitbit app on the iPad through a Bluetooth connection. After a successful upload, the study investigators could access minute-level Fitbit data through the Web-based platform, Fitabase. If a participant did not synchronize their device during the study period, a member of the study team would synchronize the device with the participant on the final day of the study during the semistructured interview session. Data from the RT-CGM devices were collected at the end of each 7-day monitoring period by synchronizing each receiver to Dexcom Studio (Dexcom Inc) on a study laptop. Food intake data were exported manually from Healthwatch 360 and Fitbit at the end of each 7-day monitoring period by a study investigator.

A semistructured interview was conducted with participants to seek insight into their experiences using the RT-CGM, the wearable activity tracker, and the mobile apps provided on the iPad. Moreover, 1 study investigator, who acted as a moderator, led the interview. The interview moderator also probed for suggestions for integration of the devices and their data in a T2DM self-management intervention. A bilingual comoderator assisted with translation of language and complex concepts if needed. The moderator led the interview using a Moderator’s Guide (see Multimedia Appendix 3), which included preselected questions and probes. The questions were adapted from the Moderator’s Guide developed by Wallen et al used in a similar community-based technology pilot study [8].

Participants were remunerated with a US $100 gift card for providing at least 1 day of reliable device data and for participating in the follow-up interview.

### Quantitative Data Analysis

Quantitative accelerometer-based data and 5-min blood glucose data were collected from all participants’ devices. All quantitative analyses were performed in R version 3.3.2 (R Core Team).

Feasibility of the devices was assessed based on device usage (ie, the higher the number of participants engaging with the devices, the higher the feasibility). Device utilization frequency for both the activity tracker and RT-CGM was measured by the number of days with device-captured data. Usage of the food logging apps was measured by the number of days with self-logged meals and the number of meals logged.

Quantitative data included steps taken, minutes of activity intensity on a 0 to 3 scale (predefined by Fitabase: 0=sedentary, 1=light, 2=moderate, 3=very active), distance traveled (measured in miles), floors climbed, calories burned (measured in kilocalories), and heart rate. For those participants with more than 7 days of data, the first full 7 days of collected data were included in the analysis. The first day of data activity and RT-CGM data were omitted from the analysis, as this information represented only a partial day and was likely not representative of a typical full day’s measurements. Days with no recorded PA data were considered *missing* and were not included when calculating average measures per day.

### Qualitative Data Analysis

Each semistructured interview was audio recorded, and the recording was translated (if needed) and transcribed by an independent Health Information Portability and Accountability Act–compliant translation and transcription service (TranscribeMe). The qualitative team comprised 4 investigators (LY, BG, ML, and NA). LY listened to the audio files to verify transcription, and all 4 members developed a codebook based on participants’ responses. In addition, 2 teams of 2 coders independently reviewed the interview transcripts and evaluated each for the presence of the codes. NVivo (version 9.0) was used for further qualitative analysis. Discordance was discussed until consensus was reached. Main themes, subthemes, and selected quotes that align with each theme are displayed in the tables to indicate important findings.

## Results

### Demographic and Clinical Characteristics

There were 21 individuals who participated in the self-monitoring period and 18 who participated in the subsequent semistructured interview and questionnaire. However, 3 participants chose not to participate in the follow-up interview and questionnaire. Participants could also choose not to answer certain sociodemographic questions (eg, income and employment). Among the participants, 76% (16/21) were female, the mean age was 50.4 (SD 11.0) years, all participants were Hispanic, and 82% (14/17) had an annual household income of <US $40,000 per year. All patients had a self-reported diagnosis of T2DM, and most (89%, 16/18) were treated with oral medications (ie, Metformin), some with insulin (17%, 3/18), and the others through diet and exercise. Demographic and clinical characteristics for the study population are presented in Table 1.

### Quantitative Data

#### Device Usage

Of the 7 possible full days of device usage, participants provided Fitbit activity readings for 6.14 (SD 0.8) days. Most participants (86%, 18/21) registered 6 days or more of activity data, with 14% (3/21) registering 3 days or less. Nearly all (95%, 20/21) participants provided RT-CGM readings, with 1 participant providing none. More than half (52%, 11/21) logged at least 1 meal in a diet tracking app, with 33% (7/21) logging meals for 4 or more days.

**Table 1:** Demographic and clinical characteristics.

Variable		Value	
**Sex (n=21), n (%)**			
	Female		16 (76)
	Male		5 (24)
Age in years (n=18), mean (SD); range		50.4 (11.0); 36-74	
**Ethnicity (n=21), n (%)**			
	Hispanic		21 (100)
**Employed (n=17), n (%)**			
	Full time		8 (47)
	Part time		5 (29)
	Retired		1 (6)
	Unemployed		3 (18)
**Annual household income (US $; n=17), n (%)**			
	<40,000 per year		14 (82)
	>40,000 per year		3 (18)
Years with T2DM^a^ (n=18), mean (SD); range		7.4 (5.2); 1-16	
Self-reported hemoglobin A1_c_ (n=13), mean (SD); range		7.42 (1.8); 5.4-11.9	
Body mass index, mean (SD); range		33.6 (6.2); 22.9-44.8	
**T2DM treatment (n=18), n (%)**			
	Medication, insulin		3 (16)
	Medication, oral		14 (78)
	Diet/exercise		10 (56)
**Type of T2DM care received (n=18), n (%)**			
	Primary care provider		15 (83)
	Specialist		7 (39)
**Has attended a T2DM education class (n=18), n (%)**			
	Yes		13 (72)
	No		5 (28)

^a^T2DM: type 2 diabetes mellitus.

#### Objective Measurements

For the days on which steps were registered by the Fitbit, mean steps per day among participants (n=21) was 7822 (SD 3984) steps. Among participants, the maximum steps per day was 11,893 (SD 5478) steps and the minimum was 4147 (SD 4218) steps. For the days on which the RT-CGM registered real-time blood glucose levels, the mean value among participants (n=20) was 148.6 (SD 47.1) mg/dL (range of participant means 94.2-247.4 mg/dL). The minimum glucose value captured among participants was 43 mg/dL and the maximum exceeded 400 mg/dL (a value of *high* is recorded when a level exceeds 400 mg/dL).

#### Technology Ownership and Usage

Nearly all survey respondents (17/18) owned a mobile phone. Of mobile phone owners, all but 1 owned a smartphone; 47% owned an Android (8/17), 41% owned an iPhone (7/17), and 6% (1/17) owned a different type of smartphone. Table 2 describes additional technology ownership and usage among the 18 survey respondents.

### Qualitative Data

The results of the qualitative study provided insight about the benefits and challenges of wearable sensors and mobile apps for T2DM self-management among a Hispanic community–based population. The analysis resulted in 3 major themes that highlight the level of acceptability of these devices: (1) advantages of T2DM self-monitoring devices, (2) user design preferences, and (3) limitations to diabetes technology use in Hispanic populations. Several subthemes were also identified and described in Table 3.

**Table 2:** Technology usage and ownership among survey respondents (N=18).

Variables			Value, n (%)
Own a mobile phone			17 (94)
Own a tablet computer			7 (39)
Own a laptop or desktop computer			9 (53)
**Email account**			17 (94)
	Daily	10 (59)	
	Weekly	4 (24)	
	Monthly	2 (12)	
	Never	1 (6)	
**Facebook account**			17 (94)
	Daily	15 (89)	
	Weekly	1 (6)	
	Monthly	1 (6)	
	Never	0 (0)	
Food logging app			1 (6)
Activity tracker on phone or watch			2 (11)
Facebook for T2DM^a^ support and education			6 (33)
Websites for T2DM support and education			7 (39)
Mobile app for T2DM support and education			1 (6)
Mobile app with glucose meter integration			1 (6)

^a^T2DM: type 2 diabetes mellitus.

**Table 3:** Semistructured interview themes and subthemes.

Themes	Subthemes
Advantages of self-monitoring devices	Device feedback supports behavior change Device feedback supports increased awareness to enhance self-management
User design preferences	Training Tailoring Comparison Social support Data sharing Data integration
Limitations to diabetes technology use in Hispanic population	Barriers to type 2 diabetes mellitus management Barriers to technology use Technology limitations

### Advantages of Self-Monitoring Devices

#### Device Feedback Supports Behavior Change

Individuals with T2DM felt that the near real-time feedback (eg, glucose levels, trends, alerts, step counts, and reminders) from the self-monitoring tools encouraged behavior modification. Most participants reported behavior modifications for selecting foods, but some participants also modified their PA behaviors or general health maintenance routine.

Most participants who reported modifying food behavior did so in response to the feedback on the RT-CGM. Examples of food behavior changes that took place included swapping a food item for a healthier choice, ending a meal prematurely if blood glucose levels were rising, or consuming carbohydrates if blood glucose levels were too low. One participant who swapped a food item for another in response to RT-CGM data noted:

Of course I have to choose. Do I eat the two tortillas or...more rice? I’m not going to eat the rice if I’m eating the—you can choose because you know it’s going to get high.

Several participants reported that they stopped eating if they saw their blood glucose rising or if the RT-CGM was delivering a *high* alert. However, 1 representative quote includes:

Sunday, we went to the buffet...I wanted to eat more to take advantage...but [the RT-CGM] didn’t let me do it. It was ring, ring, ring, and vram, vram, vibrating...I tell my husband, “I’m not going to eat anymore because this is telling me that I’m high...so I have to stop it.”

Similarly, another participant noted:

I decided to eat less or to avoid eating because my glucose level was high. Or, I was aware that I had gone too long without eating, and my glucose level was low.

Although most participants reported food modifications, some also reported changes to PA behavior because of the device feedback. Although most PA behavior modifications were in response to feedback from the Fitbit, some participants reported increases to PA behavior because of feedback from the RT-CGM. However, 1 participant reported that it helped him *make decisions to go to the gym to exercise*, whereas another reported that she chose to walk to the grocery store rather than drive when she saw that her blood glucose levels were rising on the RT-CGM receiver.

Interestingly, the feedback on the RT-CGM also encouraged participants to modify their general health maintenance and medication management regimen. Regarding the RT-CGM, 1 participant reported:

It helped me a lot to remind me to take my pill...I did not have the habit of taking my pills...But this week with the device, I was taking them constantly

Participants felt the daily use, and twice-a-day calibration of the RT-CGM was a passive reminder to meet other self-management needs. Furthermore, participants appeared most likely to modify behaviors based on the RT-CGM feedback than other devices. Patients attributed this to the novelty of the RT-CGM data and how it relayed information that they *did not already know.*

#### Device Feedback Supports Increased Awareness to Enhance Self-Management

Participants expressed that device feedback (eg, glucose levels, trends, alerts, step counts, and reminders) illuminated previously unknown relationships between certain health behaviors and T2DM measures. Some previously unknown relationships included the relationship between stress and blood glucose as well as the relationship between HbA_1c_ and glycemic variability.

Furthermore, 1 participant, in particular, recognized that on days when she did not eat breakfast, she still experienced significant blood glucose escalations that she attributed to her stress. She noted:

Yes, the stress, and I don’t eat nothing in the morning. I don’t eat nothing in the day, all day, and then “foo!.” This is new for me. The emotion affect my diabetes.

Other participants were made aware—primarily through the visualizations on the RT-CGM—of the relationship between the level and intensity of PA and changes in blood glucose levels. One participant noted that:

Even 10 minutes of walking. It does make a difference [on my blood glucose levels].

Participants who had a general knowledge of T2DM management and HbA_1c_ levels recognized that although A_1c_ values represent an average, they may be misrepresenting one’s actual glucose levels and not reflecting daily glycemic variability. One participant, who self-reported an A_1c_ value of 6.6 mg/dL, noted:

I see my sugar, after eating, it went up and then really low...My A1C is 6.6--but something is going on because my sugar is getting up more than 200. So I know what happens with your organs if it is going up more than 200. So now I can explain to my doctor what’s going on.

### User Design Preferences

#### Training

Many participants suggested that additional training and instructions would be needed for them to effectively use the self-monitoring tools in the future, especially the iPad and the food tracking apps. Many of those participants who did not use one of the devices (eg, the iPad) or used them minimally during the study reported a fear of being *nosy* or *breaking* it. Several participants reported fear of losing the iPad as the primary reason for minimal use. Despite receiving guidance and an introduction to the apps from a study coordinator, most participants were not comfortable exploring all the apps on the iPad and felt restricted to the diet tracking apps only. To alleviate this issue, participants suggested that the study coordinators should train people more extensively on *how to use [the iPad] so people know it can go not only on the food one [application]* *but on the other ones.*

#### Tailoring

Participants felt that a future T2DM self-monitoring intervention leveraging these tools would be most effective if it was tailored to the individual patient. One participant felt that the diabetes technologies should be tailored differently to different members of the Hispanic community. He noted that:

Because they speak the same language does not mean that they are the same. They value things differently...You want to say Hispanic community? Foreign-born or US born? 18-22 are totally different than 30-50.

Similarly, participants raised the concern of delivering a technology-facilitated intervention to a population of varying technology and health literacy. One participant noted:

Some are not literate enough that they could use a computer or even a cell phone to text. So not to expect a 70 year old, all 70 year olds to be at the same level. It’s just not going to happen.

In addition to tailoring to cultural and sociodemographic characteristics, most patients felt that they would be most likely to engage with a future intervention that was tailored to time and meal. For instance, 1 participant remarked positively about the time-based reminders on the Fitbit at mealtimes and after long periods of sedentary time but would have preferred more specific advice or goals. An example she provided was:

So it would be lunch time and [it could remind you] “Don’t forget your salad! Don’t forget your veggies!” You’re like, “Oh my goodness. Need to do it.”

Other participants expressed that not all patients have the same needs, and some may require diabetes specialty providers who can provide more specific counseling related to nutrition or medication management. They felt that a technology-facilitated T2DM intervention should cater to these varying types and levels of support. One participant explained this in the following scenario:

Look, I need more specialized help--like nutrition, or maybe an endocrinologist to look at [my data] to actually say, “this is what is happening to you.” Maybe, my general doctor or physician might not be able to provide me good feedback [but an endocrinologist can].

#### Comparison

The Dexcom G4 visualizations are restricted to a small window that shows blood glucose trends for only a few hours at a time. Many participants expressed that the RT-CGM would have been most beneficial if they were able to compare their glucose over time, whether that was between days or weeks. One participant noted that:

When I was checking the graphic, I thought I can scroll more for the next day, the day before and the day before and no. It’s only for one day.

Participants desired a comparison feature to ensure that any modifications they made while self-monitoring were leading to improved outcomes. One participant said:

So what I want to do in myself is improvement. Compare one week and say, “This was the first week.” But I need to see the change in the second week, and another progress in the third, like that...And at the end, keep those good habits...But I need to see the change.

#### Social Support

Use of the self-monitoring tools required a certain level of positive social support. Participants felt that the tools—as standalone devices—were not enough to promote behavior change. Rather, they desired peer, family, community, and medical support complementary to the devices. Most felt that, with support, the self-monitoring tools could aid in improving HbA_1c_ levels. One participant noted:

If you want to bring my A1c down from 8 to 5, and this would help me monitor my diet and everything, and I do it with medical support, and hopefully peer support, now that is pretty darn good. Now, you’re talking about something that I am willing to do.

Some participants also desired peer support specifically from others with T2DM, as they would have an *idea of what you can do or the type of activity that would require you to go from 200 to 160 mg/dL.*

The highest percentage of participants desired and relied on technical, emotional, and/or behavioral support from a spouse or family member. Several participants brought family members to the follow-up interviews, and most noted that a family member also engaged with the devices at some point during the study period. Several participants wanted the ability to share their data in real time with their spouse and felt that doing so was, at times, more important than sharing with a health professional. One participant noted:

If [your spouse] knows, they are closest to you, they can support and/or correct whatever. It’s a good thing.

However, not all spouse support was positive. A few participants noted the emotional stress that self-monitoring tools can place on a spouse or family member. One participant shared:

I can see a point of view that [the RT-CGM] can be really stressful. For my husband it was...He said, “You know what? Just turn it off. I don’t want to hear it. I don’t want to hear it if you have your sugar level up or down. Don’t do it. It’s making me crazy.”...So I mean, I can see his point there.

#### Data Sharing

Participants expressed the need to have the ability to share their T2DM device data with health professionals or a health coach to receive interpretation of the data, guided feedback, or personalized advice through messaging. Many participants also expressed the need for controlling which data were shared and with whom. One participant noted:

There are programs that have been developed that say, “I shared all my data with my doctor.” But, I would only share this data with my [health] coach. Then, you, as my [health] coach can actually enter if you have my permission. You can go into the program and say, “Okay, you’re doing great.”...As a coach, if I’m working with 10 people, then I could have access to the information that they have agreed to provide me. Then I can provide better feedback on a constant basis. Then, I could text them.

#### Data Integration

Participants felt overwhelmed with multiple devices and multiple streams of data and expressed a need to view their data in once place. To alleviate this burden, 1 participant recommended that the study team consider the *consolidation of all the artifacts into 1 single place.* The recommended medium for visualizing the streams of data was on a smartphone. One participant commented:

It’s better if I can have [everything] in my phone. It’s with me. I sleep with that.

Participants desired a single device that was already part of their routine. Participants also recommended integrating different sources of data such as T2DM-related measures from the electronic health record. One participant commented that having health measures, such as recent HbA_1c_ values, is *something ideal to be more in control.*

### Limitations to Diabetes Technology Use in Hispanic Populations

#### Barriers to Type 2 Diabetes Mellitus Management

Our results revealed that T2DM knowledge, lifestyle, and willingness to engage in self-management, among others, existed as barriers to adequate T2DM management. Several participants did not understand the expected effect of medications such as Metformin. Some even mentioned taking Metformin to lower their blood glucose when they were experiencing hyperglycemia. One individual reported the following scenario:

Last night, [the RT-CGM] was showing 189 after I ate the hamburger, so...I went home and I took the—I don’t take too much medicine because I don’t want it—so I took medicine, and then I want to take another one, my husband said, “No, don’t take another one.” Because it was 189. And [the RT-CGM] was ta-ta-ta-ta-ta-ta.

Other participants reported thinking that the RT-CGM was not working because they were not observing a change in their blood glucose trend on the RT-CGM after taking their prescribed dose. One participant stated, “I take Metformin medication *...* and the [RT-CGM] does not reflect it.” These quotes suggest a misunderstanding related to the mechanism of action of the medication and how that would be reflected in the CGM data.

In addition to T2DM-related knowledge, busy lifestyles remain a primary barrier to patients’ T2DM behavior change. Some participants reported being parents with multiple children and holding several jobs and felt that making healthy food choices was difficult; therefore, they preferred options that were easy, inexpensive, and accessible. One woman recalled a recent scenario:

I got to my house, I have broccoli, I bought a bunch of broccoli. I don’t want it. I’m not in the mood for broccoli. I’m hungry. I’m shaking, I’m hungry. Yeah, quick, fast.

Other participants expressed that motivation and willingness to self-manage one’s T2DM are also likely barriers. One individual recounted:

Before, I would not have been glad [to participate], because one wants to eat what one wants to eat. I am trying to educate myself [about diabetes], and that is why I liked [these tools] because I already have that in my mind.

#### Barriers to Technology Acceptance and Use

Most participants expressed that main barriers to use were fear, trust, calibration requirements, comfort, and cost. Participants were most fearful of the small insertion site for the RT-CGM. One participant commented:

I talked...some of my co-workers and my family too, about the [RT-CGM] that I have. They said, “Oh, it’s nice. I would like to have one of those, but I’m scared of [the insertion].”

Other participants were fearful that the device would move while in use; therefore, they restricted their activity during the study period. One participant commented:

I don’t make exercise this week because I scared about [the CGM] moving or something...Only walking, that’s it.

Another participant raised the concern about trust. He experienced glucose fluctuations when comparing his self-monitoring of blood glucose (SMBG) level with the RT-CGM level when calibrating the device. As a result, he felt unsure about which value was accurate. He commented:

Which one do I trust? This thing is plugged into my body, and I would think that the measurement of this item is more accurate than the one that I’m doing in my finger. Yet, the discrepancy between the two readings at times was quite significant. And, I thought, okay which one is correct. Here the machine says I’m good, but my finger says not.

Calibration posed additional challenges for participants. Often, participants did not calibrate the device in 12-hour intervals twice a day as recommended. Some participants also reported forgetting the receiver during the day, which results in an inability to transmit data.

Comfort was also cited as a deterrent to the use of the self-monitoring tools, particularly irritation from the rubber wristband of the Fitbit and the adhesive on the RT-CGM. One participant whose sensor pod and transmitter became unattached to her insertion site reported discontinuing use of the RT-CGM during the study period because it *broke*. She said:

The truth is, if it didn’t break I would have used it every day, but it was very fragile. I don’t believe it would have been able to break.

Another participant discontinued RT-CGM use and removed the transmitter and sensor pod after 4 days due to discomfort at the insertion site.

When asked about the use of these tools for self-monitoring in the future, most participants cited cost as a barrier. Those participants without health insurance commented that these devices would be out of reach. However, after being told the cost, some participants were willing to purchase the device. One participant mentioned that if such a tool would prevent him from being on insulin, he would buy one. He commented:

Before I go to insulin, do you think that I wouldn’t be happy to pay $300/month for 6 months [for sensors]? Oh, heck yeah. I don’t want to get into insulin. If my doctor after seeing this says you need to get into insulin. Nope. Just simply, I won’t. So, this would be a good way to say, “Okay, let’s work together on this.”

#### Technology Limitations

Certain technology limitations restricted the optimal use of the T2DM self-monitoring tools. These included proficiency with SMBG, insulin adjustments based on RT-CGM levels, and the language offerings on the RT-CGM and the iPad apps (ie, lack of Spanish).

The RT-CGM required daily calibration and previous exposure to SMBG; however, a few participants were not accustomed to daily SMBG, and 1 participant had never practiced SMBG at all. This required extra training by the research team for participants to be prepared to calibrate the RT-CGM daily during the self-monitoring period.

Several participants felt that the user interface of the RT-CGM was specific to insulin-requiring diabetes and not to those who are not insulin requiring. They felt the options for inputting insulin dose and the number of carbohydrates consumed were confusing. One participant stated*:*

So, this assumes that I take Insulin, but I don’t. I take pills. So, that seems to me, one piece that is lacking. 

Furthermore, no apps or tools used in the study were offered exclusively in Spanish, posing a challenge for nearly all participants. The RT-CGM was limited to English only, making it more challenging for those who primarily spoke Spanish, limiting usability related to features that used the menu (ie, calibrations). Food logging was especially challenging. One participant noted that:

There is a lot of Latino foods that you guys already have in there, but...you’re missing a bunch.

## Discussion

### Principal Findings

The objective of this study was to engage Hispanic community members with T2DM in the evaluation of the feasibility, acceptability, and potential integration of self-monitoring technologies and their data into a future self-management intervention. Both quantitative and qualitative findings revealed that the tools—used in a relatively unguided manner—successfully captured and communicated T2DM self-monitoring data (ie, food intake, PA, and blood glucose level) on most days. Although participants were inexperienced in using the 3 self-monitoring tools at baseline, nearly all used both the RT-CGM and the activity tracker to self-monitor, and more than half logged food intake on the iPad. Strengths and weaknesses of the T2DM self-monitoring devices and the user experience as well as preferences for integration and data sharing were identified and categorized into themes, which will inform the design of an acceptable, technology-facilitated T2DM self-management intervention targeting this group.

This study is among the first to explore the use and potential integration of multiple T2DM self-monitoring technologies in a community of low-income, primarily middle-aged Hispanic adults with T2DM. Our findings indicate that using a multimodal system (comprised a RT-CGM, a wearable activity tracker and a digital food diary) is not only feasible and acceptable but also educational, as it unveils previously unknown relationships between lifestyle and health and contributes to changes in T2DM-related behaviors among *both* insulin-requiring and noninsulin-requiring patients with T2DM (in clinical practice, RT-CGM is offered primarily for insulin-requiring patients). Our findings also highlighted that a successful T2DM self-management intervention leveraging multiple devices and their data should be offered on a single device; should be accessible and actionable to patients, clinicians, and family members; and should be tailored to the unique needs of the individual user. In the community group targeted in this study, providing culture-specific content was also a critical need.

### Multimodal System for Type 2 Diabetes Mellitus Self-Monitoring on 1 Device

In an era characterized by the internet of things, sensors for capturing multiple health behaviors, exposures, and symptoms are ubiquitous [9]. Minimizing the burden of multiple sensors and devices in health interventions is critical for patient engagement. In our study, patients interacted more with the devices that required less user input—the RT-CGM and the Fitbit—and less with the tool that required more input—the iPad for diet tracking. This is consistent with studies on self-tracking, where tools that require additional time and attention see a lower level of engagement [10]. In our study, individuals noted that the iPad was burdensome to use outside the home because of its size and fear of losing it. Participants suggested consolidation of the tools and visualization of the device data, on a smartphone, for instance, as an avenue for minimizing user burden. Nearly all participants in this study reported owning a smartphone and cited a smartphone as a preferred platform for viewing self-monitoring data. Technology companies, such as Fitbit and Dexcom, are beginning to answer this need through partnerships and the integration of health apps on a single smartphone and smartwatch platform [11].

### Interoperable System for Facilitating Support From Peer or Professional Care Team

Although patients felt generally favorable about the standalone tools used in this study, they desired a data sharing feature to facilitate support from a family, peer, or professional care team. Health care partnerships have been cited previously as a key element for engaging and retaining users of self-monitoring digital health tools in the clinical setting [12,13]. Patients have reported a lack of confidence in their ability to synthesize various self-monitoring data streams and cite a care team as a missing piece of this puzzle for additional support [12].

An additional patient need, as identified in previous studies, also included control of access to self-monitoring data. Our participants desired functionality that allowed them to choose with whom they shared their data. Patient choice on *who* and *when* to share data with care partners has been cited as a critical consideration in RT-CGM data sharing [14]. Including a care partner in one’s data sharing has been shown to enhance patients’ perception of safety [14].

Although most of the literature point to data sharing with a health care team, our participants also highlighted the need for preferential data sharing with a peer health coach or community health worker. Previous work has shown that peer support interventions for patients with T2DM are most effective for those have difficulty engaging with T2DM, have inadequate access to T2DM support, and have lower levels of health literacy [15]. In our study, patients desired to have a peer health coach be the first line of support and provide them with access to certain data, reserving other data for providers to access. Peer support interventions, specifically those that involve community health workers, are not uncommon in diabetes care, and they have shown a clinically significant effect on glycemic levels and diabetes-related behaviors, knowledge, and self-efficacy among Hispanic with T2DM [16].

### Actionable Insights and Feedback for Facilitating Behavior Change

After just 7 days of device use, several patients found meaning in their data, which led to both increased awareness of how behaviors affect their blood glucose level and self-reported behavior change. In particular, participants reported the RT-CGM was helpful in making behavior changes related to food and exercise behavior. This supports findings in other CGM studies.

We did not provide participants with a priori instructions on actions to improve their glucose readings, but in a similarly designed study, participants took immediate actionable steps to solve blood glucose excursions without any self-management education [17]. The potential for CGM technology in individuals with T2DM to make clinically significant changes is great. After only wearing the CGM for 6 weeks over a 3-month period, participants had decreased their HbA_1c_ level by 1%, and their HbA_1c_ remained significantly decreased at 0.8% at 12 months [18,19]. Although participants in this study were not provided self-management education, individuals were able to draw on past experiences and receive insight from educational resources or trained professionals.

Although participants valued the device feedback, particularly that from the RT-CGM, they desired enhanced feedback that was actionable and contextual, such as built-in personalized advice on lowering blood glucose level in real-time or historical context comparing current state to a past state. As referenced in a similar study on digital health tools [12], this need for insight and context supports the theory of *sensemaking*, suggesting that people can make predictions on behavior change based on information gathered from past experiences and then derive meaning about their present actions and environment [20,21]. However, little is known about what (if any) RT-CGM features (eg, trend arrow, current glucose value, and profile from past few hours) guide decision making [22]. Studies have shown that patients are not using their downloaded RT-CGM data to make decisions, but rather using data presented on the RT-CGM device itself [23]. In our study, not all participants derived meaning from their data, and some who derived did not draw accurate conclusions.

These additional features that support sensemaking should be offered on self-monitoring devices in real-time to help patients understand their data and draw accurate conclusions that lead to behavior change and increased awareness. Recent work has shown that simulated data demonstrating the acute effect of PA on blood glucose may even be enough to change PA-related outcome expectancies and behavioral intentions among adults with T2DM [24].

### Cultural Tailoring

Our study is one of the first to explore the needs of Hispanic adults using T2DM self-monitoring technologies. To date, T2DM self-monitoring apps and devices targeting limited English proficiency Hispanic communities are lacking. Although all participants in our study were relatively proficient in English, nearly all preferred content on the devices be delivered in Spanish. Few apps on the iPad were available both in Spanish and at a recommended reading level for patient education, a finding consistent with the recent literature [25]. To overcome this barrier for food tracking, Fitbit offers different food databases from which participants can choose. Dexcom also offers content in Spanish (eg, user guide), although the G4 receiver used in this study was not available in Spanish for participants. Since our study, flash glucose monitors, such as the Freestyle Libre (Abbott Diabetes Care, Inc) and its integrated smartphone app, have joined the market and received Food and Drug Administration approval [26]. Flash glucose monitors are a sensor-based hybrid between RT-CGMs and glucose meters. They measure blood glucose levels throughout the day and provide access to the readings by scanning the sensor. This device suite may address the language barrier presented by the Dexcom, as it provides both a user guide and smartphone app available in Spanish. Despite the noted benefits, this 14-day flash glucose monitoring system does not provide real-time continuous access to glucose levels as the Dexcom suite does. To access glucose levels, the sensor *must* be manually scanned, and an iPhone app facilitating this process has recently been developed. Although a recent meta-analysis demonstrated that RT-CGM was effective at reducing the HbA_1c_ level, the evidence to promote the effectiveness of flash glucose monitoring in individuals with T2DM was not conclusive [27]. The lack of significant HbA_1c_ reduction may be the result of the lack of real-time data and alerts. However, the significant cost difference between the continuous glucose monitoring and the more affordable flash glucose monitoring suites may make this a target for future study in the Hispanic population.

### Strengths and Limitations

Strengths of this study include the use of T2DM self-monitoring tools targeting a low-income, racial and ethnic minority group, the incorporation of CBPR strategies, and the combination of both qualitative and quantitative data gathered during the pilot test. In addition, our study investigated primarily *unguided* use of multiple digital health tools, allowing us to determine likely barriers in the real-world rather than in an idealized setting. Most participants voiced a desire to use the RT-CGM and the Fitbit for longer periods of time, suggesting that the tools were valued and accepted and would observe similar success in a long-term study.

Limitations of this study must also be acknowledged. The study was limited to the 7-day lifespan of the RT-CGM sensor. This limited our ability to adequately test participant participation with the research protocol, engagement, and retention over time. Future work would benefit from a study 30 days or longer to understand how participant engagement changes over time, although we believe that a 7-day study period was adequate to understand major facilitators and barriers to use. Generalizability may also be a concern. Our sample size consisted of 21 participants, all Hispanic and from different countries of origin, but now living in the same geographical region—a region to be targeted by a future T2DM intervention. This study sample is not an adequate representation of a particular foreign-born or US-born Hispanic population. Future work would benefit from extending this study to a larger and more diverse sample to better evaluate if country of origin or years in the United States contribute to certain aspects of T2DM self-management. In addition, individuals in this study had various experiences with T2DM. Ensuring that all participants have experience in SMBG in future work would aid in improving accuracy and ease of calibration of the RT-CGM. Finally, no standard score for measuring health literacy or average English proficiency was used. Future studies would benefit from standardizing these measurements to better characterize our population of interest.

Owing to our focus on the unguided use of these tools, training was limited. Future studies exploring these tools would benefit from in-depth training and direct contact during the 7 days to troubleshoot potential technological difficulties. A past community-based technology study has suggested creating a community point-person or *super user* who understands the technology and the needs of the community and can troubleshoot during the research period [8]. Including tools available in both English and Spanish would aid in uptake among the community, although in the case of any digital health study, researchers are limited by the availability and offerings of existing tools.

### Conclusions

Sensor-based tools for facilitating T2DM self-monitoring appear to be a feasible and acceptable technology among a low-income, Hispanic community–based population in Utah. Community-based methods, particularly pilot testing and semistructured interviews, aided in early identification of issues and user preferences for the future design of a technology-facilitated T2DM intervention. We identified barriers to acceptability and highlighted preferences for wearable sensor integration. These findings have implications for the design of T2DM interventions targeting racial and ethnic minorities. Additional work is needed to understand how to guide patients in decision making using their device data and visualizations. Although feedback from devices aids in enhancing an individual’s awareness, insight that is actionable and personalized is likely needed to promote sustained behavior change. Of equal importance is understanding the implications of integrating T2DM self-monitoring tools and their data into the clinical setting. Although patients desire the integration of T2DM self-monitoring tools, careful consideration of a care team’s needs will be critical to their success. Future work will investigate the use of T2DM self-monitoring data to drive a simulation-based, problem-solving intervention that highlights problem areas, suggests opportunities for improvement, addresses facilitators and barriers to behavior change and guides the participant in goal setting.

## Appendix

Multimedia Appendix 1 Mobile apps (with descriptions) offered on iPad.

Multimedia Appendix 2 Self-monitoring device guide.

Multimedia Appendix 3 Moderator's guide for semistructured interview.

## Abbreviations

CAB: community advisory board
CBPR: Community-Based Participatory Research
HbA_1c_: hemoglobin A_1c_
PA: physical activity
RT-CGM: real-time continuous glucose monitor
SMBG: self-monitoring of blood glucose
T2DM: type 2 diabetes mellitus
