# Supplementary material for: An Evaluation of Digital Health Tools for Diabetes Self-Management in Hispanic Adults: Exploratory Study
Source: JMIR Diabetes. 2019 Jul 16;4(3):e12936. doi: 10.2196/12936 (PMC6664655; doi:10.2196/12936)
Supplement: Multimedia Appendix 1 [file diabetes_v4i3e12936_app1.docx]

**Multimedia Appendix 1**

| **Application** | **Description** |
| --- | --- |
| Headspace | Stress reduction with guided meditations and mindfulness techniques |
| Fitbit | Tracking for all-day activity, workouts, sleep and more. Can connect with Fitbit activity trackers to get a complete picture of health—including steps, distance, calories burned, sleep, and weight |
| Healthwatch360 | Dietary logging and nutrition goal setting. Provides daily reports on nutrition status, monitors 30+ nutrients in foods consumed and allows for tracking exercise and health symptoms |
| Es Tu Diabetes | Spanish social network for diabetes support and education |
| Diabetes Detective | Goal setting and problem solving for optimizing blood glucose levels |
| Fooducate | Network for nutrition support and education and database of healthy recipes |
| CanopySpeak | Translation service for patient/provider interactions |
