# Supplementary material for: An Evaluation of Digital Health Tools for Diabetes Self-Management in Hispanic Adults: Exploratory Study
Source: JMIR Diabetes. 2019 Jul 16;4(3):e12936. doi: 10.2196/12936 (PMC6664655; doi:10.2196/12936)
Supplement: Multimedia Appendix 2 [file diabetes_v4i3e12936_app2.docx]

**Multimedia Appendix 3**

Dear __________________,

Thank you for participating in the PCORI Technology Pilot Test. By participating, you are agreeing to complete an online anonymous survey and wear and/or use 3 different mobile devices for one week. At the end of the 7-day trial period, we will have a follow-up session. At this session, we will collect the devices, conduct a short 30-minute interview with you to understand your experience with the devices, and give you a gift card for your time.

The second page has a list of the items you will be receiving and details on each.

Your **follow-up session** is:

**Date:**

**Location:**

1. Continuous Glucose Monitor (CGM)

- Sensor + Transmitter (inserted below the skin)
- Receiver
- Charging Cord (black) + Charging Port (black)
- *****REMEMBER*****
  - ***
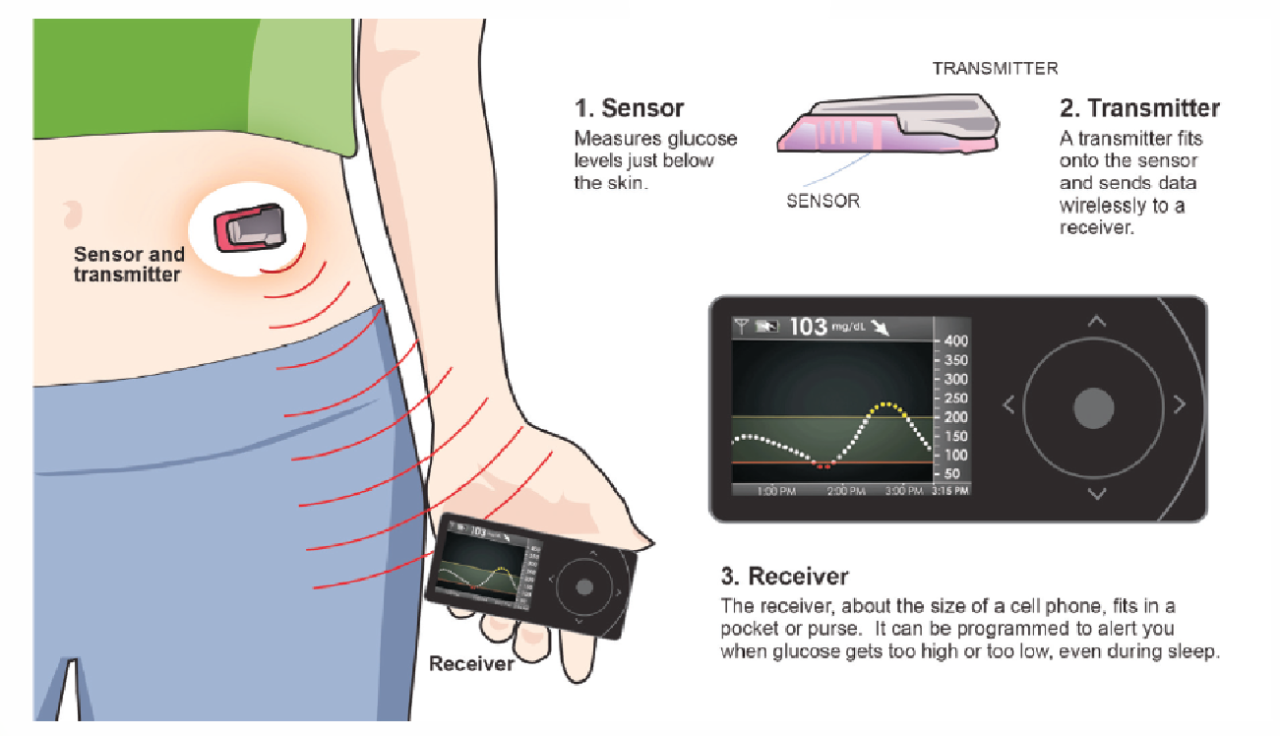
CALIBRATE DEVICE IN THE MORNING & NIGHT.***
  - ***KEEP RECEIVER WITH YOU AT ALL TIMES.***

***
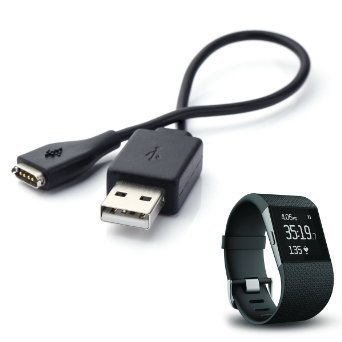
***

1. FitBit Surge
   - Wristband
   - Charging Cord (black)
   - *****REMEMBER*****
     - ***WEAR AT ALL TIMES***
     - ***REMOVE IF SUBMERGING IN WATER (BATHING/SWIMMING)***
     - ***REMOVE IF IRRITATION OCCURS***
2. iPad
   - iPad
   - Charging cord (white)
   - Charging cube (white, can be used to charge FitBit too)
   - *****REMEMBER*****
     - ***USE ANY OF THE IPAD APPS FOR EDUCATION AND SELF-MANAGEMENT***

***
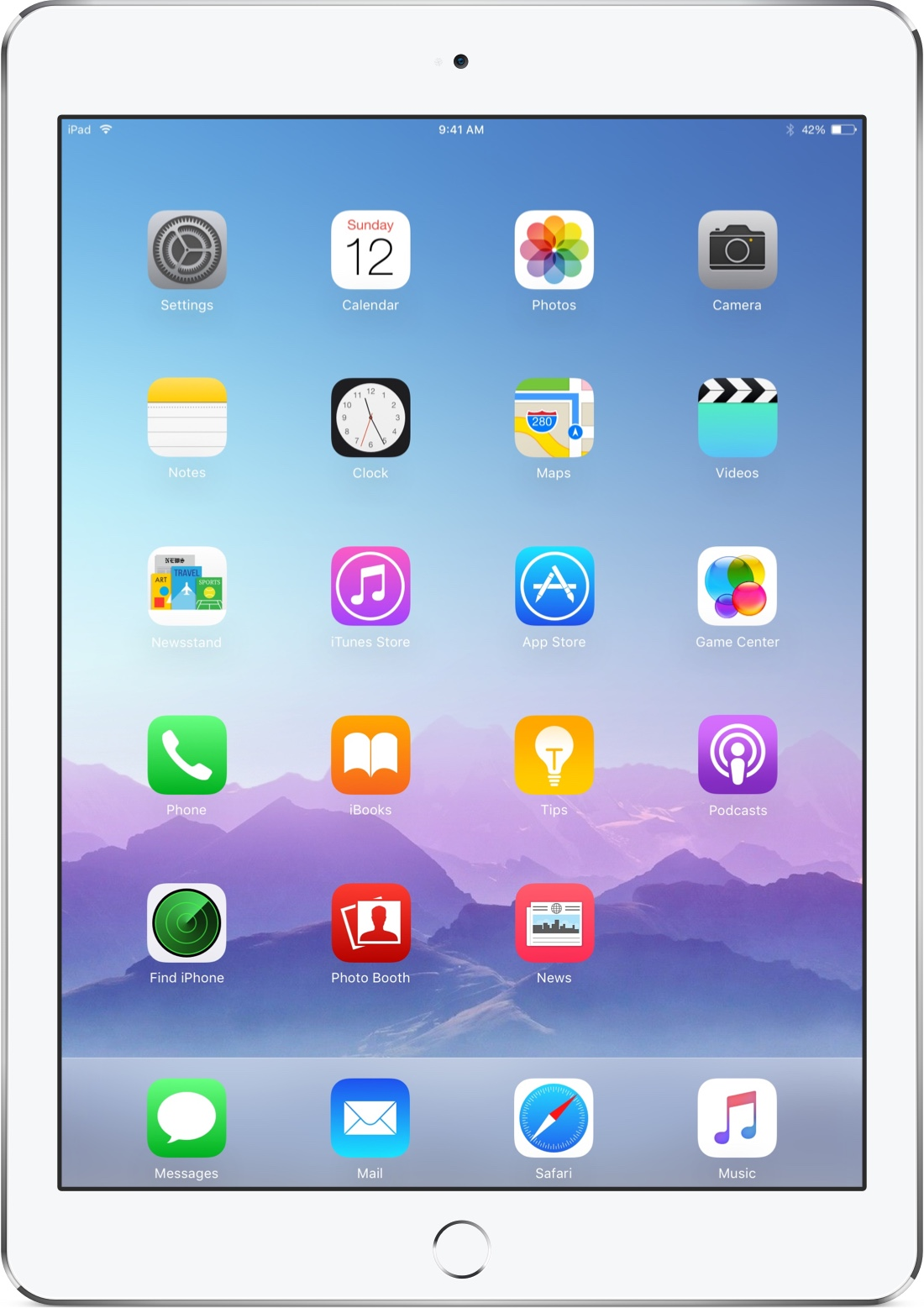
***
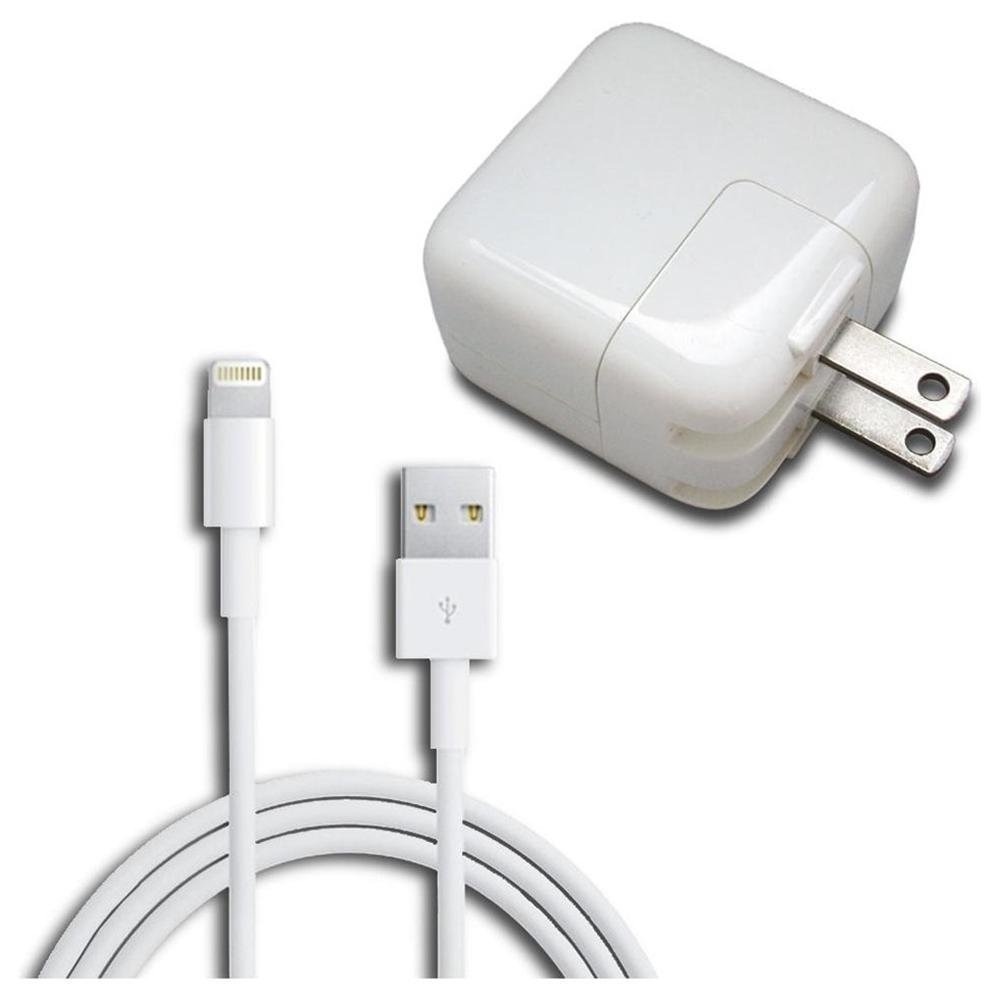
**What we are asking you to do:**

- Wear the **FitBit** every day and use any iPad apps that you’d like.
- Log **everything** you eat on the iPad **FitBit app** every day. Please select below your preference:
  - I will log everything I eat once a day @ _______.
  - I will log everything I eat twice a day @ _______ & ________.
  - I will log everything I eat right away.
- Check your blood glucose with your glucose meter twice/day and calibrate your CGM with the values.
  1. Morning @ _______ A.M.
  2. Night @ ________ P.M.
-
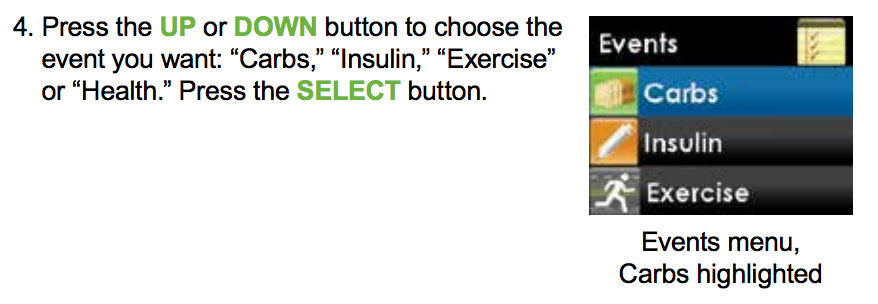

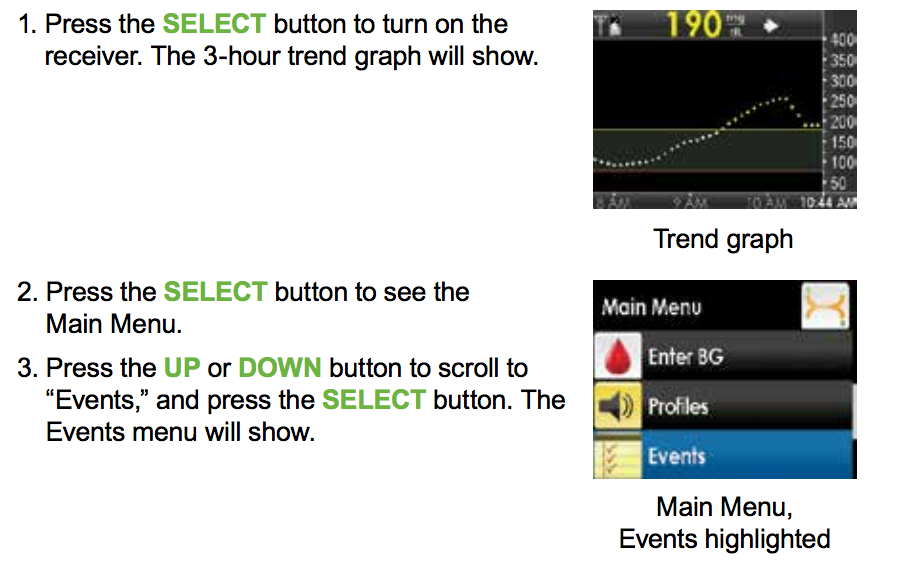
Use the CGM to mark each time you eat

1. Choose **Carbs** and press the **SELECT** button
2. It will default to “50 grams.” Press the **SELECT** button to confirm.
   - **We don’t need you to select the exact number of carbs you are eating, we just want to know **when** you are eating, so **do not** select the exact number of carbs you are eating. Just select 50 and press the **SELECT** button.**
