# Supplementary material for: An Evaluation of Digital Health Tools for Diabetes Self-Management in Hispanic Adults: Exploratory Study
Source: JMIR Diabetes. 2019 Jul 16;4(3):e12936. doi: 10.2196/12936 (PMC6664655; doi:10.2196/12936)
Supplement: Multimedia Appendix 3 [file diabetes_v4i3e12936_app3.docx]

**Multimedia Appendix 2**

**ONE-ON-ONE SEMI-STRUCTURED INTERVIEW**

**MODERATOR’S GUIDE FOR**

**PCORI TECHNOLOGY PILOT TEST**

*(Adapted from Moderator’s Guide developed by Dr. Gwenyth Wallen, RN, PhD^1^)*

1. **One-on-one interviewing session**
   - We will thank participants for cooperating in the one-week technology pilot test.
   - We will explain to participants the purpose of the session regarding their experience using the FitBit for tracking physical activity, the continuous glucose monitor for monitoring blood glucose values, and the iPad apps for logging diet and self-managing their diabetes.

*NOTE: We will be audio-recording during the individual sessions to ensure that all feedback and information exchange is collected. Each session will last up to thirty minutes.*

1. **Key Questions

   A. Continuous Glucose Monitor (CGM)***REMEMBER: Continue to probe after each question to get the participant’s perspective on the use of the device.*
2. Do you have previous experience using a CGM?
   1. If yes, please tell me about your previous experience.
3. Did you have any problems using the CGM?
   1. If yes, could you tell me about the problem?
4. What features of the CGM did you like best?
5. What features did you not like about the CGM?
6. How many times/day did you check your readings?
7. Did you have any skin irritation, rash, pain or physical issues while wearing the device?
   1. If yes, can you tell us about those issues?
8. Did you experience any issues calibrating the device two times a day (once in the morning and once at night)?
9. When you calibrated the device, did you ever see a large fluctuation in your glucose meter reading and the CGM reading?
10. This device is $1,198 and 1 month sensor supply is $349, would you buy one of these for yourself?
11. Would you wear this if your insurance paid for it?
12. Did wearing this change how you think about your diabetes?
    1. If yes, please explain.
13. Did wearing this change your behaviors (diet, physical activity medication adherence)?
    1. If yes, please explain.
14. If you could have “shared” your continuous data with others would you have done this?
    1. What are you other thoughts about this feature?
    2. If you could do this, who would you share your data with?
15. Would you share this data anonymously with other people with diabetes to help them learn more about their diabetes?
16. Any other thoughts/ comments?

**B. FitBit Activity Tracker**

1. Do you have previous experience using a wearable activity tracker?
2. Did you have any issues collecting or viewing your physical activity data in the last week?
3. Did you look at the wristband to check your data during the day?
   1. If so, approximately how many times did you do this?
   2. What type of data did you check the most (sleep, steps, hr)?
   3. What type of data did you check the least?
4. Did you have any skin irritation, rash, pain or physical issues while wearing the FitBit?
   1. If so, can you tell us about thos issues?
5. Did wearing the Fitbit encourage you to increase your own physical activity and exercise more often?
6. What features on the FitBit did you like?
7. What features did you dislike?
8. Would you buy one of these for your own?
9. Did wearing this change how you think about your diabetes?
10. If you could have “shared” your data with others, would you have done this?
    1. If you could do this, who would you share your data with?
11. Any other thoughts/ comments?

**C. The iPad**

1. Do you have previous experience using an iPad?
2. Have you used health-related apps on an iPad before
3. Were there apps that you used regularly this past week?
   1. If so, which ones were they?
   2. What did you like best about these?
4. Were there apps that you never used this past week?
5. How many times a day did you look at the apps?
6. Do you plan on using any of these apps in the future?
7. Did using these apps change how you think about your diabetes?
8. If you could, would you be willing to share information collected by these apps with anyone (e.g., dietary intake, meditation, goals, etc.)?
   1. If so, who?
9. Any other thoughts/ comments?

**D. Integration + Future**

1. These apps and devices all work a bit separately right now, how would you like to see them work together?
2. In a perfect world what functions would you like to see these devices or apps to provide? (If not sure, prompt with prediction, recommendations, etc.
3. If you wore these devices on a regular basis for 3 months, would you like a health coach to call to provide personalized diabetes education using information from these devices?
   1. If yes, what would you like about this?
   2. If no, why not?
4. Would you like the diabetes health coach to help you with other problems that get in the way of taking care of your diabetes such as problems affording your diabetes medicine, supplies, accessing health care, taking your medicine, legal problems, social and mental health problems?
   1. If yes, which of these types of support would you prefer?

**E. Wrap-up**

1. Do you think people in your community would use these devices?
2. What do you see as potential barriers for people using these devices in your community?

**F. Closing**

- After the final question, the moderator would again thank the participant for taking part in the interview.
- The Moderator will inform the participant that the feedback will be a part of a process of designing the best way to use tools for diabetes self-management in an intervention
- The moderator will remind the participants about confidentiality in discussion of this study with others.
- Participants will receive remuneration for their participation in this interview.

**G. References**

[1] Leah R Yingling, Alyssa T Brooks, Gwenyth R Wallen, Marlene Peters-Lawrence, Michael McClurkin, Rebecca Cooper-McCann, Kenneth L Wiley Jr, Valerie Mitchell, Johnetta N Saygbe, Twanda D Johnson, Rev. Kendrick E Curry, Allan A Johnson, Avis P Graham, Lennox A Graham, Tiffany M Powell-Wiley. JMIR Mhealth Uhealth 2016 (Apr 25); 4(2):e38
